# Recent developments and potential of robotics in plant eco-phenotyping

**DOI:** 10.1042/ETLS20200275

**Published:** 2021-05-20

**Authors:** Lili Yao, Rick van de Zedde, George Kowalchuk

**Affiliations:** 1 Wageningen University & Research, Wageningen, Netherlands; 2 Universiteit Utrecht, Utrecht, Netherlands

**Keywords:** eco-phenotyping, phenotyping, plant monitoring, robotics

## Abstract

Automated acquisition of plant eco-phenotypic information can serve as a decision-making basis for precision agricultural management and can also provide detailed insights into plant growth status, pest management, water and fertilizer management for plant breeders and plant physiologists. Because the microscopic components and macroscopic morphology of plants will be affected by the ecological environment, research on plant eco-phenotyping is more meaningful than the study of single-plant phenotyping. To achieve high-throughput acquisition of phenotyping information, the combination of high-precision sensors and intelligent robotic platforms have become an emerging research focus. Robotic platforms and automated systems are the important carriers of phenotyping monitoring sensors that enable large-scale screening. Through the diverse design and flexible systems, an efficient operation can be achieved across a range of experimental and field platforms. The combination of robot technology and plant phenotyping monitoring tools provides the data to inform novel artificial intelligence (AI) approaches that will provide steppingstones for new research breakthroughs. Therefore, this article introduces robotics and eco-phenotyping and examines research significant to this novel domain of plant eco-phenotyping. Given the monitoring scenarios of phenotyping information at different scales, the used intelligent robot technology, efficient automation platform, and advanced sensor equipment are summarized in detail. We further discuss the challenges posed to current research as well as the future developmental trends in the application of robot technology and plant eco-phenotyping. These include the use of collected data for AI applications and high-bandwidth data transfer, and large well-structured (meta) data storage approaches in plant sciences and agriculture.

## Introduction

**Figure 1.**
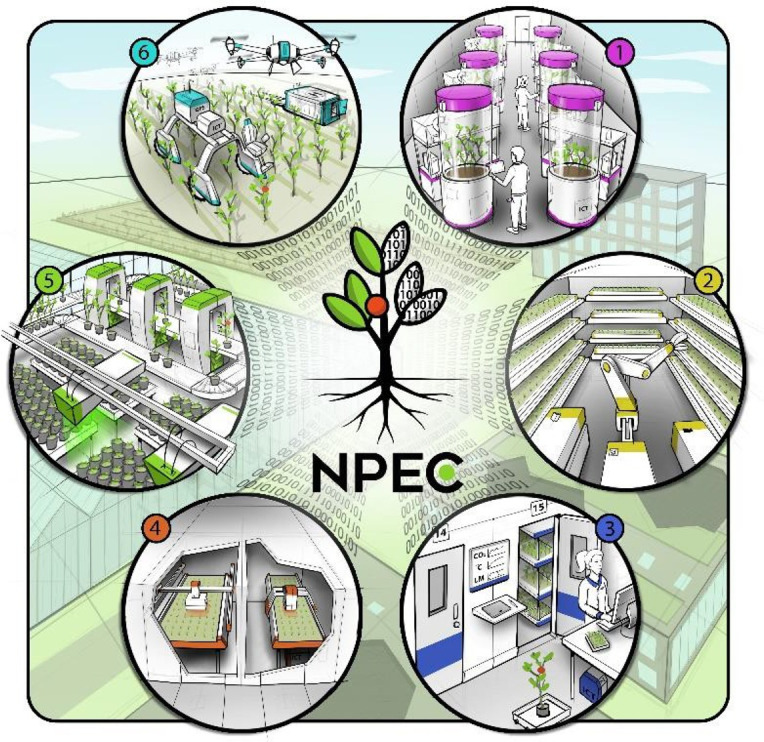
Visualization of the six modules in the Netherlands Plant Eco-phenotyping Centre (NPEC) [9].

Measuring plants with sensors, plant phenotyping, was originally defined in the field of crop breeding as the biological traits, structure, size, color, and other expressions *in vitro* determined by the genotype and the environment [1]. With the continuous development of digital phenotyping research, the conceptual category has been linked to the fields of biochemistry, molecular biology, and behavior [2–6]. However, both the micro-level and macro-level plant phenotyping information have an inseparable relationship with the ecological environment. Individual plant development is also influenced by interactions with (neighboring) plants, microbes, other organisms, and a more realistic examination of plant performance should therefore include the effect of biotic interactions on the plant phenotype. Examples include studying the role of the plant microbiome; the bacterial and fungal populations that colonize plants both above- and belowground. Plants in the ecological environment have various ways to respond to changes. Phenotyping changes are a concrete manifestation of the response of plants to the environment [7]. To deepen the relationship between phenotyping information and ecological environment and make it more targeted, researchers from the Netherlands Plant Eco- phenotyping Centre (NPEC) first proposed the term *eco-phenotyping* and defined it as plant phenotyping under ecologically relevant conditions. Ecological conditions mainly include biotic (microbiome interactions, competition, disease) and abiotic factors (light quantity and quality, nutrients, temperature, moisture, soil pH, and atmospheric CO_2_ level) [8]. In response to the concept of eco-phenotyping, they are also carrying out a series of eco-phenotyping facility construction plans, as shown in Figure 1. The metadata information precisely describing environmental information is also critical to link between observed phenotypic variation and genotypic variation versus environmental differences.

There are many kinds of potential plant phenotyping parameters, and the efficient acquisition of these phenotyping parameters can provide effective information to support agricultural production management. Traditional monitoring methods mostly rely on manual measurements, which shows a low accuracy, poor efficiency, and limited acquisition of additional metadata, making it difficult to meet the demand of modern big data applications [10,11]. To improve the efficiency of obtaining plant phenotyping information, a range of robotic platforms has been used in plant phenotyping monitoring research. These robot platforms have the characteristics of flexible movement and a high degree of automation. Such systems can replace many human inspection tasks to achieve semi-automatic or even fully automatic operations. The combination of robotic platforms and high-precision phenotyping monitoring sensors (various RGB, multi- and hyperspectral cameras, 3D-sensors [12], etc.) has further advanced the ability to enhance the complete study of plant for eco-phenotyping. In addition, Artificial Intelligence (AI) technologies, such as deep learning, big data mining, and machine learning, provide the means to process and interpret plant data which was collected via high- throughput devices [13–16].

This article provides an overview of the application of existing robot technology in plant eco- phenotyping monitoring. We conclude with a discussion of potential bottlenecks and shortcomings of current research efforts by summarizing the role of the current application. Agricultural robot technology is rapidly developing, and its combined application with AI technology and 5G communication technology opens up a vast range of possibilities. A major challenge in the research field will provide the insight on how to best take advantage of emerging technological possibilities within the agri-food and research settings.

## Sensors and sensing technologies in eco-phenotyping

**Figure 2.**
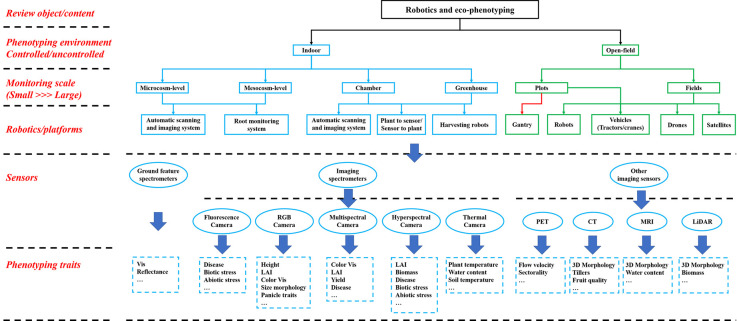
The figure of robotic platforms, phenotyping sensors, and phenotyping parameters used in plant eco-phenotyping.

Phenotyping monitoring sensors and related sensing technologies are an important basis for plant eco-phenotyping. In recent years, with the development of ground feature spectral monitoring technology, spectral monitoring equipment has been utilized more in plant eco-phenotyping applications and have realized real-time, non-destructive, rapid, and efficient plant phenotyping monitoring [17–20]. According to different perception principles, these sensors mainly have ground feature spectrometers, spectral imaging sensors, and other imaging spectrometers, as shown in Figure 2.

**Figure 3.**
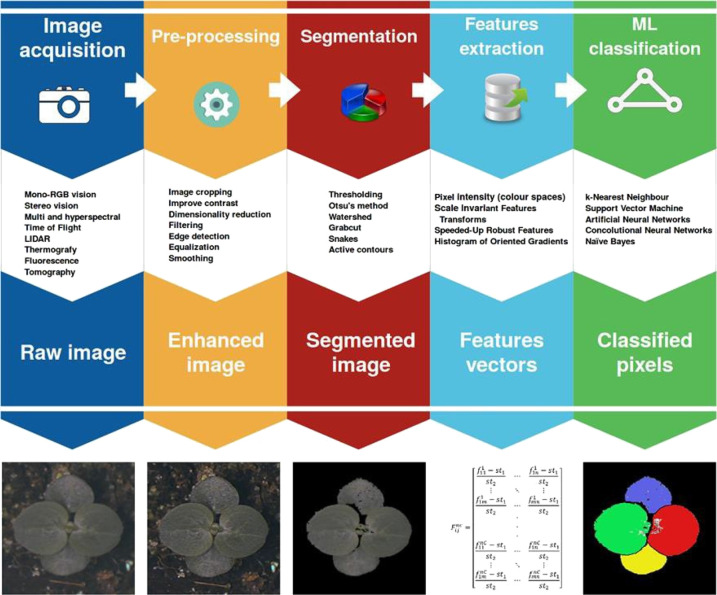
The basic workflow in imaging sensor-based plant phenotyping [38].

Ground feature spectrometers can use photodiodes, optical fibers, and other photoelectric sensing devices to collect the spectral reflectance of the crop canopy at specific wavelengths and calculate some vegetation indices to achieve phenotyping parameters inversion. Since the beginning of the research on the ground feature spectrometer, some commercial instruments that provide accurate results have been widely used [21–30]. Spectral imaging sensors can be used to obtain spectroscopic images of a specific waveband that contain more information than a ground feature spectrometer, the basic workflow in imaging sensor-based plant phenotyping is shown in Figure 3. According to the difference of the spectral band of the acquired image, spectral imaging sensors include RGB cameras, multi-spectral cameras, hyperspectral cameras, fluorescence cameras, thermal cameras, etc. [31–37].

In addition to spectral imaging, non-spectral imaging technologies such as Light Detection And Ranging (LiDAR), Computed Tomography (CT), Positron Emission Tomography (PET), Magnetic Resonance Imaging (MRI), and related equipment have also gradually gained popularity for the acquisition of phenotyping information [39–43]. Regarding the specific imaging principle and the acquired phenotyping parameters, Lei Li et al. [44] have published more detailed results. Artificial Neural Networks (ANN), Support Vector Machine (SVM), Convolutional Neural Network (CNN), Recurrent Neural Network (RNN), and other AI algorithms have been widely used in the study of phenotyping information acquisition as well as analyses such as phenotyping parameters measurement, feature recognition, and disease detection [45–50].

## Construction of indoor robotic and eco-phenotyping platforms

Plant eco-phenotyping monitoring scenarios can be divided into two major categories that are eco-phenotyping in the indoor environment and eco-phenotyping in the open field. As ecological factors are relatively controllable within indoor settings, eco-phenotyping monitoring was first applied within indoor environments. Relying on the effective combination of robotics and automatic environmental control technology, the high-throughput phenotyping monitoring platform TraitMill^TM^ was introduced [51]. It's used in the plant-to-sensor working mode, which can automatically obtain the phenotyping information of potted plants in the greenhouse. Since then, scientific research institutions across the world have successfully launched a range of phenotyping platforms for indoor phenotyping information acquisition [52,53]. However, most of these automated robotic platforms focus on the observation of plant macroscopic phenotyping parameters, such as plant height, leaf area, leaf color, etc. With the further development of plant science and electronic technology, research and exploration of plant phenotyping have gradually developed to the micro-scale [54]. According to the different observation scales of phenotyping information [55,56], the current indoor plant eco-phenotyping monitoring platforms can be divided into four monitoring levels: Greenhouse-level, Chamber-level, Mesocosm-level, and Microcosm-level.

Greenhouse-level plant eco-phenotyping mainly relies on the plant-to-sensor model or sensor- to-plant model [57]. With the former model, the plant moves to the sensor test point for observation through an automatic transmission mechanism [58–60]. The latter realizes the observation of different plants by moving the position of the sensors [61,62]. Although their goals are realized in two different ways, these are still considered as reliable approaches in automatic monitoring through robotic technology. Gravimetric systems have shown the capacity to observe plant performance and behavior from a different angle by analyzing water usage, evaporation rate, and related parameters. Such approaches can provide an insight about more subtle plant phenotypic traits and examine responses to imposed stresses such as drought and salinity [63]. As compared with other scales indoor plant eco-phenotyping, Greenhouse-level systems provide sufficient planting dimensions to allow the acquisition of phenotyping information throughout the full lifecycle of the plant. This can facilitate a range of studies in plant breeding, growth monitoring, and pest monitoring studies, with the distinct advantage of allowing examination of late growth-stage elements of plant phenotype such as maturation characteristics. A number of mobile robot platforms and manipulator structures have been designed to accommodate a range of crops and these platforms use phenotyping monitoring sensors to analyze the phenotyping information. while these platforms are also developed to measure ripeness levels of fruits for automated for picking and classification [64–66].

**Figure 4.**
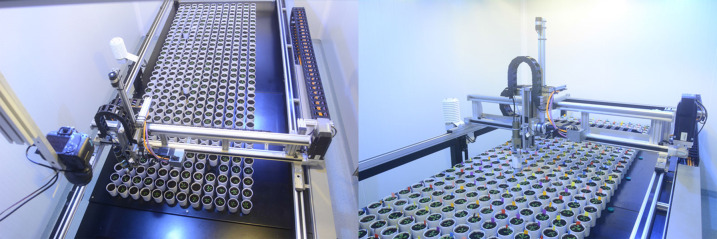
WIWAM XY plant system (https://www.wiwam.be/phenotyping-systems/wiwam- xy/) [72].

Chamber-level eco-phenotyping is similar to Greenhouse-level systems concerning phenotype monitoring sensors, automation technology, and robot technology [67,68]. They, however, differ in cultivation area and monitoring scale. While Greenhouse-level systems cover a large area but often fail to achieve accurate control of most ecological factors. Chamber-level systems use relatively small rooms that can achieve accurate control of temperature, water, CO2, light conditions, disease infection, and other biological and abiotic stresses. This allows for more accurate quantification of the plant eco-phenotyping traits that appear in response to highly specified and controlled environmental conditions [69,70]. To this day, there have been many successful projects utilizing Chamber-level plant eco-phenotyping based on high- precision and high-throughput robot platforms, such as the WIWAM XY plant phenotyping system [71], as shown in Figure 4.

**Figure 5.**
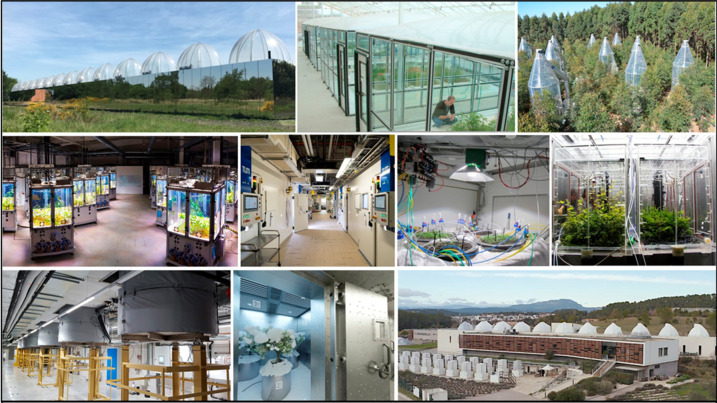
Ecotrons systems over the world [38,73].

Mesocosm-level ecological phenotypic studies help to simulate the ecological environment and facilitate microbial community studies, allowing both internal and external biological properties to be measured without penetrating the ground. By studying physiological responses exhibited by plants when the plants were interacting with either conspecific or interspecific neighbors, the ecological footprint of cropping systems provide insight into the development of more eco-friendly cropping strategies [73]. Rhizotrons are one of the earliest non-destructive underground Mesocosm-level platforms. Limited by the available sensor technology, early rhizotron facilities typically used cellars or underground corridors with transparent glass on both sides. Researchers can walk through the facilities and directly observe the root phenotyping and soil conditions in-depth underground [74]. However, these underground facilities are expensive, difficult to maintain, destructive to soil structure, and not conducive to large-scale use [75]. The further development of sensor technology, improvement of agricultural cultivation techniques, and advances in soil sensors with high integration have all facilitated the engineering of small volume systems with a high degree of accuracy — so-called mini-rhizotrons [76]. Researchers can monitor root phenotypes and soil information more accurately by embedding sensors in the soil. Thanks to the advanced sensor technology, the researchers can achieve higher monitoring efficiency and cost reduction. Ecotrons represent another type of Mesocosm-level platform that attempt to simulate an even larger scope of environmental integration. Ecotrons refer to replicated, enclosed experimental systems that aim to replicate realistic environmental conditions both above- and belowground, while also measuring a range of ecosystem processes. In addition to monitoring soil and plant root phenotypes, Ecotrons can also simulate various natural environmental conditions to not only better monitor the influence of different ecological environment factors on plant phenotypic information, but also to track the impact of such factors across a range of ecosystem parameters [77,78], Econtrons systems used in current research are shown in Figure 5.

Eco-phenotyping studies at the cellular and molecular levels can provide researchers with insights about the mechanical relationship between ecology and phenotype. For this reason, phenotyping information monitoring at the Microcosm-level has emerged as a research hotspot in recent years [79]. A range of approaches has been utilized to gain fine-scale information related to plant physiological responses to environmental conditions. For instance, Magnetic Resonance Imaging (MRI) has been used to obtain structural information related to physiological processes in plants [80]. Targeted cell and plant organ structure analysis have been conducted by using Photoacoustic Tomography (PAT) [81], and High-speed Confocal Microscopy and High-content Screening Systems have been used to study the genetic mechanism of plant disease resistance [82–84].

## Robotics and eco-phenotyping in open field settings

Unlike the plant eco-phenotyping in the indoor environment, the ecological conditions in the open field cannot be accurately controlled, causing various interference factors. Outdoor settings generally have the advantages of comprising a larger cultivable area where there is hardly any housing infrastructure. It is possible to acquire phenotyping information of large-scale crop populations through remote sensing technology. To improve the efficiency of obtaining plant phenotyping information in an open field, a range of robotic platforms has been designed. According to the scale of information acquisition, these platforms can be divided into proximal sensing platforms, low-altitude remote sensing platforms, and high-altitude remote sensing platforms.

Proximal sensing platforms are mainly used for phenotyping monitoring sensors to obtain the phenotyping information from an individual plant or groups of plants with above ground tissue of >3 m[78,85]. Proximal sensing platforms enable to obtain high resolution information with a great level of precision. However, the test efficiency is lower as compared to low-altitude and high-altitude remote sensing systems due to the small test range. As a result of the different cultivation methods and growth characteristics of different plants, proximal sensing platforms used in required customization, and development facilitated to the specific crop and uncertain cropping system, to avoid the destruction of plants in the process of operation [52,86,87]. In addition, proximal sensing platforms are often used in agricultural harvesting because of their proximity to the plants. In addition to carrying phenotyping monitoring sensors, aforesaid systems can be equipped with agricultural harvesting machinery such as manipulators and vibration rods [88,89].

Low-altitude remote sensing is mainly carried out through drone platforms or some robot platforms such as Gantry or Crane with sufficient height and extension capabilities [90,91]. Compared with Proximal sensing platforms, commercial drone technology has become more mature. Drone technology in general is a domain of rapid advance, spurring on the development of a decent range of agricultural remote sensing drone products [92,93]. In addition to advances in drone technology, it is equally important that development-oriented research of new phenotyping monitoring sensors, novel analysis tools and in-depth analysis of acquired data is achieved [45,50,94].

High-altitude remote sensing is often carried out by satellites or Unmanned Aerial Vehicle (UAV) platforms. With the increasing demand for agricultural monitoring, the design of satellite sensors has been gaining significant research attention. By analyzing medium resolution satellite images or high-resolution satellite images, crop classification, crop phenotyping information, crop biophysical and chemical parameters can be effectively extracted. The commonly used satellite series include the Sentinel-1/2 satellite, WorldView satellite, and Landsat satellite, etc. [95–97]. For more detailed information for the specific application of satellite images, the reader is referred to the recent overview on this subject published by Chongyuan Zhang [98].

## Future perspectives

With the recent acceleration of agricultural modernization designed to meet increasing food demands, considerable efforts and capital have been invested in the more systematic and precise development of modern crops and cropping systems. To this end, precision eco-phenotyping systems offer a range of possibilities to stimulate the necessary advances in these fields. The range and precision of sensor devices are developing rapidly, so as the sophistication and applicability of robotic platforms. Together, these developments provide a quantum leap in opportunities for the improvement of phenotyping and phenomics technologies. Phenotyping is the key to understand how plants perform within a given ecological setting. The collection and analysis of phenotyping data should ultimately support crop breeding, cultivation practice, and agricultural management. However, there are still some obstacles in the current phenotyping and phenomics studies.

First of all, the existing technologies have been relied on a certain proven range of sensor devices to obtain a variety of phenotyping information. However, efficient methods that combine different sensors and imaging technologies into a practical application process are still developing. The analysis of different sensor data also relies on different hardware devices, software systems, and analysis methods. Such complex operation process slows down data acquisition and integration, leading to an information lag. In addition, even regarding the application of the same sensor device, it is also difficult to combine the data obtained due to the different platforms and different monitoring scales used, which highlights the need for international harmonization and standardization [99]. The advent of the 5G or even 6G eras should facilitate the ability for rapid data transfer and interconnectivity between platforms. High-throughput data no longer needs to be stored in on-site storage devices but can be processed in cloud-based platforms in real-time. Breaking through the bottleneck of information transfer, the operation process of data acquisition, transmission, and storage management can be significantly simplified. The construction of a new data exchange platform also means that the global plant eco-phenotyping data will be stored in a common same public domain. The further development of different sensor data processing and analysis technology is also expected.

Secondly, AI technology is currently experiencing an unprecedented global research boom. Plant eco-phenotyping is also one of the important fields of AI application. Whether it is through machine vision or deep learning to process plant phenotyping information, or to apply these technologies to robotic platforms to make them more intelligent, AI will further promote developments in phenotyping and phenomics [12]. In current AI research, the researchers typically pay attention to constructing efficient AI systems for phenotyping information acquisition. F Perez-Sanz et al. [38] gave a more detailed overview of the combined application of AI technology and image acquisition technology in plant phenotyping and cited a large number of examples. We suggest that the future application of AI technology should focus on cooperation and communication between different systems. For example, with AI technology as a linking tool, phenotyping monitoring, plant breeding, crop cultivation, and agricultural management can be brought together.

Thirdly, the collection of eco-phenotypic data, from both plants and their environments, offers the opportunity to provide an actual feedback loop to virtual models of plants, cropping systems, and growth conditions. This new n combination, also named digital twin, will require the combination of multiple areas of expertize [100]. Relevant models like the functional-structural plant (FSP) model [101,102] have been made to simulate individual plants and their functioning (e.g. photosynthesis) as well as their 3D architectural development based on a set of physical and physiological plant parameters. With eco-phenotypic data and automation, it is becoming feasible to develop a very advanced digital twin concept, in which a simulation model predicts growth in 3D, yield, use of nutrients, water, CO2, and energy for multiple crop varieties. Such analyses can also examine the profit and environmental impact by using real- time phenotypic measurements of the plants and environmental growing conditions. Based on the predictions and data collected by the eco-phenotyping tools and robots, model parameters can be aligned with the sensor data, for instance, there might be a case for the new cultivar that gives a different growth response to temperature or some other parameters. In this approach, growth management can be optimized, and genotype selection can also be supported for breeding applications.

In conclusion, there are great new possibilities, and these require integrated approaches which demand to apply biotic and abiotic factors around the plant into the experiments. Furthermore, the ambition should be not only on measurement and sensors only, but eco-phenotyping should also take the responsibility to include the associated data management aspects, and make sure the community gets full access to all data, parameters, source code and metadata.

## Summary

- Plant eco-phenotyping monitoring can effectively reveal the relationship between ecological environmental factors and plant phenotyping performance. Robotic platforms have diverse shapes, flexible movement, and efficient operations that can be used in different plant eco-phenotyping settings and scenarios, thereby accelerating the modernization and digitalization of plant eco-phenotyping monitoring.
- Due to the relatively controllable environmental factors, indoor plant eco-phenotyping and robotic platforms preceded outdoor platforms. Through high-precision sensor equipment and intelligent data analysis methods, the current research has a good understanding of indoor plant macro and micro phenotyping changes.
- Outdoor plant eco-phenotyping is subject to less human intervention conditions, and the phenotyping changes of plant individuals and groups are closer to the natural conditions. Simultaneously, outdoor conditions are more conducive to aerial robotic platforms, which can obtain plant population data with a higher spatial resolution to observe the population effect on the individual plant phenotyping.
- In the future construction of a plant eco-phenotyping and robotics, the design of robotic platforms should be combined with AI technology, so that it has higher flexibility and versatility and can adapt to the test of various plants. The data combination analysis method between robotic platforms with different monitoring scales also needs to be developed. Ultimately, the application of higher-speed communication technology in this field should not only brings faster data transmission but also further promotes the construction of global plant eco-phenotyping Internet of Things (IoT).

## Abbreviations

AI: artificial intelligence
MRI: Magnetic Resonance Imaging
NPEC: Netherlands Plant Eco-phenotyping Centre
